# Impact of Different Hydrate Forms of Magnesium Stearate as a Flow Control Agent on the Physical Stability and Inhalation Efficiency of Carrier-Based Formulations

**DOI:** 10.3390/pharmaceutics17060711

**Published:** 2025-05-28

**Authors:** Jin-Hyuk Jeong, Jaewoon Son, Ji-Hyeon Kwon, Chang-Soo Han, Chun-Woong Park

**Affiliations:** 1 Department of Pharmacy, Chungbuk National University, Cheongju-si 28644, Republic of Korea; jinddong92@gmail.com (J.-H.J.); kwonjihyun1201@gmail.com (J.-H.K.); 2 GC Biopharma, MSAT/DP Team, Yongin-si 16924, Republic of Korea; sonjw96@gccorp.com; 3 P2KBio, Cheongju-si 28160, Republic of Korea

**Keywords:** magnesium monohydrate, hydrate form, force control agent, aerodynamic performance, carrier based dry powder inhalation

## Abstract

**Objectives:** This study aimed to evaluate the impact of the different hydration states of magnesium stearate (Mg.st) anhydrate (AH), monohydrate (MH), and dihydrate (DH) on the aerodynamic performance and stability of carrier-based dry powder inhalation (DPI) formulations using arformoterol and budesonide as model drugs. **Methods**: DPI formulations were prepared using Inhalac 251 lactose and Mg.st in various hydrated forms. The physicochemical properties of Mg.st were characterized using powder X-ray diffraction, differential scanning calorimetry, Fourier-transform infrared spectroscopy, Karl Fischer titration, dynamic vapor absorption, and Raman imaging. The aerodynamic performance was assessed employing a next-generation impactor under initial and accelerated conditions (40 °C, 75% relative humidity). **Results**: Mg.st-MH exhibited the highest crystallinity and the most stable moisture sorption profile, and showed the smallest particle size within the formulation as observed in the Raman images. Formulations containing Mg.st-MH demonstrated significantly higher fine particle fractions for both arformoterol (51.02 ± 5.16%) and budesonide (61.98 ± 4.09%) compared to formulations with Mg.st-AH or -DH forms. Mg.st-MH also exhibited improved performance retention under accelerated conditions, correlating with its physicochemical stability. **Conclusions**: The monohydrate form of magnesium stearate was the most effective force control agent, which reduced interparticulate interactions, thereby enhancing the inhalation efficiency and formulation stability. Thus, selecting an appropriate hydration form of Mg.st can improve DPI performance.

## 1. Introduction

Dry powder for inhalation is an attractive drug delivery system for the treatment of pulmonary diseases. Compared to other inhalation formulations, such as pressurized metered-dose inhalers, soft mist inhalers, and nebulizers, dry powder inhalation (DPI) formulations are propellant-free, portable, and physicochemically stable [1,2]. In DPI formulations, the fluidization, disaggregation, and dispersion of the powder formulation are achieved solely through the patient’s inspiratory actions [3]. The characteristic properties of DPI formulations a critical role in ensuring effective therapeutic efficacy [4].

The therapeutic efficacy of DPIs is largely dependent on their aerosolization efficiency and the amount of drug deposited in the lungs [5,6]. Good powder flowability enhances manufacturability, improves fluidization, and minimizes excessive retention within the device [7]. However, a known paradox exists in DPI formulations wherein drug particles typically need to be sized between 1 and 5 μm for efficient deep lung deposition, yet such fine particles tend to exhibit high cohesiveness and poor flowability, which can hinder dispersion and delivery efficiency [8,9]. To address this issue, larger carrier particles, such as lactose, are commonly employed to improve the flowability and dispersibility of the drug powder within the DPI formulation [10].

Various formulation strategies have been developed to mitigate interparticulate interactions, including the modification of particle morphology, size, and surface characteristics, such as roughness [11,12,13]. A relatively simple approach involves the use of ternary mixtures containing fine lactose to occupy high-energy binding sites on the carrier surfaces [14,15]. Another widely used strategy is to incorporate force control agent (FCA) materials with low surface free energies during blending [16].

Magnesium stearate is a well-known excipient used not only in DPI formulations but also in oral solid dosage forms to improve powder flow. Depending on the manufacturer, Mg.st is available in various hydration states, including anhydrous, monohydrate, dihydrate, and, in rare cases, trihydrate forms [17,18]. The degree of hydration of Mg.st can influence its lubricating properties in tablet formulations [18,19,20]. However, limited data are available regarding how the different hydration states of Mg.st affect inhalation performance in DPI systems.

This study aimed to evaluate the impact of different magnesium stearate hydrate forms on the aerosolization performance of lactose carrier-based dry powder inhaler (DPI) formulations. Two model drugs with differing carrier–adhesion–balance (CAB) characteristics were selected: arformoterol, a representative long-acting β₂-agonist (LABA) with high cohesive force, and budesonide, a representative inhaled corticosteroid (ICS) with strong adhesive interaction with lactose [21]. Moreover, the stability of these formulations under accelerated conditions was examined to assess the influence of Mg.st hydration forms on DPI performance and highlight their relevance in the formulation of effective carrier-based inhalation products.

## 2. Materials and Methods

### 2.1. Materials

Arformoterol tartrate and budesonide were provided by KOREA UNITED PHARM (Seoul, Republic of Korea). Inhaled 251 cells were obtained from MEGGLE (Wasserburg, Germany). Magnesium stearate anhydrous (Mg.st-AH) was supplied by FACI Asia Pacific (Jurong Island, Singapore), magnesium stearate monohydrate (Mg.st-MH) was obtained from Daejung (Siheung, Republic of Korea), and magnesium stearate dihydrate (Mg.st-DH) was provided by PETER GREVEN (Venlo, The Netherlands). Three hydroxypropyl methylcellulose capsules were obtained from Suheung (Cheongju, Republic of Korea), and acetonitrile (HPLC grade) was purchased from Honeywell Burdick & Jackson Ltd. (Muskegon, MI, USA). All other chemicals were of analytical grade and were used as received. All experiments were performed using Milli-Q distilled water.

### 2.2. Physicochemical Properties of Different Magnesium Stearate Hydrate Forms

#### 2.2.1. Powder X-Ray Diffraction

The powder X-ray diffraction (PXRD) patterns of different Mg.st hydrate forms at 0, 1, and 2 weeks under acceleration conditions were measured using SmartLab (Rigaku, Tokyo, Japan) with Cu Kα radiation generated at 40 mA and 40 kV. The samples were placed on a silicon plate at room temperature, and 2*θ* scans were collected from 5° to 90°.

#### 2.2.2. Fourier Transformation-Infrared Spectroscopy

Fourier transformation-infrared spectroscopy (FT-IR) was performed on different Mg.st hydrate forms at 0, 1, and 2 weeks under acceleration conditions in the range of 500–4500 cm^−1^ using a Cary670 (Agilent, Santa Clara, CA, USA).

#### 2.2.3. Differential Scanning Calorimetry

The thermal properties of different Mg.st hydrate forms were analyzed using differential scanning calorimetry (DSC) (Q2000^®^, TA Instruments Ltd., New Castle, DE, USA). The samples were heated from 20 °C to 250 °C at a rate of 10 °C/min under a nitrogen flow of 50 mL/min.

#### 2.2.4. Water Content

The water content of the different Mg.st hydrate forms was measured using a Karl Fischer 901 Titrando with an 803 Ti stand (Metrohm, Herisau, Switzerland). Approximately 10 mg of each sample was placed in the instrument, and the moisture content was subsequently measured using a volumetric method. The moisture content was calculated using the following equation Equation (1):

(1) $$Water\;content\;\left(\%\right)=\frac{Amount\;of\;water\;detected\;(mg)}{Sample\;wieght\;(mg)}*100$$

#### 2.2.5. Particle Size Distribution

Different Mg.st hydrate forms were dispersed in air for particle size distribution (PSD), which was measured using a Mastersizer 3000 (Malvern, Worcestershire, UK).

#### 2.2.6. Dynamic Vapor Absorption

The moisture sorption profiles of each magnesium stearate type were determined using dynamic vapor absorption (DVS; DVS Intrinsic, Surface Measurement Systems Co. Ltd., London, UK). Approximately 10 mg of each sample was placed into the sample cell and exposed to incremental increases in relative humidity (RH), in 5% steps from 0% to 95%, until the weight stabilized to three decimal places for at least 1 min at each step. DVS measurements were conducted at 25 °C.

### 2.3. Preparation of Lactose Carrier-Based Dry Powder Formulation Using Arformoterol and Budesonide with Various Types of Magnesium Stearate

Arformoterol and budesonide formulations were prepared using various types of magnesium stearates, as shown in Table 1. To minimize drug adhesion to the inner surface of the glass bottle, the carrier—magnesium stearate—and drug were weighed in a specified order and placed in a glass bottle. The components were pre-mixed at 2000 rpm for 5 min using a multitube vortex mixer (Hangzhou Miu Instruments, Hangzhou, China). The resulting mixture was sieved through a No. 35 sieve (500 μm), followed by post-mixing under identical conditions (2000 rpm, 5 min) using a multi-vortexer to obtain the final dry powder inhalant.

### 2.4. High Performance Liquid Chromatography Analysis

Analysis was performed using the Thermo Ultimate 3000 system (Thermo Fisher Scientific, Waltham, MA, USA) with a 5 μm C18 column (100 Å, 250 mm × 4.6 mm) from GL Sciences (Tokyo, Japan). For arformoterol analysis, the mobile phase consisted of acetonitrile and 0.1% perchloric acid solution (30:70, *v*/*v*) at a flow rate of 1.0 mL/min. The column temperature was maintained at 35 °C, and the injection volume was set to 20 μL. The mobile phase also served as the diluent. For budesonide analysis, a mixture of acetonitrile and pH 3.9 buffer solution (50:50, *v*/*v*) was used as the mobile phase, with a flow rate of 1.0 mL/min. The column temperature was controlled at 50 °C, and the injection volume was 20 μL. A 30% acetonitrile solution was employed as the diluent for sample preparation. Both analyses were performed at a detection wavelength of 240 nm.

### 2.5. Aerodynamic Performance

The aerosol performance of carrier-based formulations with the Hamihaler device (Hanmi, Seoul, Republic of Korea) was evaluated using a next generation impactor (NGI, COPLEY Scientific, Nottingham, UK) following the USP Chapter <601> specifications for aerosols. To evaluate the effect of different magnesium stearate hydrates on inhalation performance under accelerated conditions, samples were stored openly at 40 °C and 75% relative humidity for 1 and 2 weeks. A flow rate of 60 L/min was set and confirmed before each experiment using a flowmeter (DFM 2000; COPLEY Scientific, Nottingham, UK). The NGI stage collection plates were coated with silicone oil to prevent particle bounce and re-entrainment during the test. Number 3 HPMC capsules were manually filled with 20 mg of the prepared formulations.

The mouthpiece was mounted on an induction port, and the devices were inserted into it. The experiments were performed at a flow rate of 60 L/min for 4 s, controlled utilizing a flow controller (TPK-R^TM^, COPLEY Scientific, Nottingham, UK). The amount of the drug remaining in the capsule and deposited onto the collection plate in each state was quantified using a modified HPLC method. Aerodynamic cut-off diameters of each stage were determined as 8.06 µm, 4.46 µm, 2.82 µm, 1.66 µm, 0.94 µm, 0.55 µm, and 0.34 µm for stages 1 to 7 at a flow rate of 60 L/min. The emitted dose (ED) was defined as the percentage difference between the initial weight and remaining weight of the drug in the capsule after aerosolization. The fine particle fraction (FPF) was defined as the ability of a particle to reach the respirable region with an aerodynamic size of approximately 5.0 μm or less. FPF is expressed as the proportion of the ED collected at stage 2 through the micro-orifice collector (MOC). This is based on the following Equations (2) and (3):

(2) $$Emitted\;dose\left(ED\right)\%=\frac{The\;total\;dose\;in\;capsules-Drug\;amount\;remaining\;in\;the\;capsule\;and\;devices}{The\;total\;dose\;in\;capsules}*100$$

(3) $$Fine\;particle\;fraction\left(FPF\right)\%=\frac{Total\;drug\;amount\;on\;stages\;2\;through\;MOC}{Total\;drug\;amount\;on\;all\;stages}*100$$

Mass median aerodynamic diameter and geometric standard deviation were calculated from the drug mass deposition during the NGI stages. All experiments were performed with n = 3.

### 2.6. Raman Image of Formulations with Different Magnesium Stearate Hydrate Forms

A random-scanning confocal Raman microscope (Ramanwalk, Nanophoton Corporation, Osaka, Japan) equipped with a 532 nm diode laser was used to acquire individual Raman reference spectra from pure components and Raman image data from powder mixtures. The powder samples were gently flattened onto glass microscope slides to ensure a smooth and uniform surface. To compare the spatial distribution and composition of the components in each formulation, Raman imaging was conducted on single particles prepared in the same manner as the actual formulations.

The interactions between arformoterol, different MgSt hydrate forms, lactose monohydrates, and ARF-Mg were visually investigated. St-AH, ARF-Mg.st-MH, and ARF-Mg.st-DH were prepared and subjected to multiparticle Raman imaging analysis. A total of 80,442 spectra were collected at a step size of 1 μm, with the neutral density filter set at 180. Raman images were generated using the direct classical least-squares method, and spectral acquisition was completed in approximately 23 h. In the resulting Raman images, red indicates arformoterol, blue corresponds to magnesium stearate, and green represents lactose monohydrate particles.

### 2.7. Statistical Analysis

All statistical analyses were conducted using a one-way ANOVA with GraphPad Prism 8 (release 8.4.2; San Diego, CA, USA). *P*-values smaller than 0.05 were considered statistically significant.

## 3. Results and Discussion

### 3.1. Physicochemical Properties of Different Mg.st Hydrate Forms

**Figure 1:**
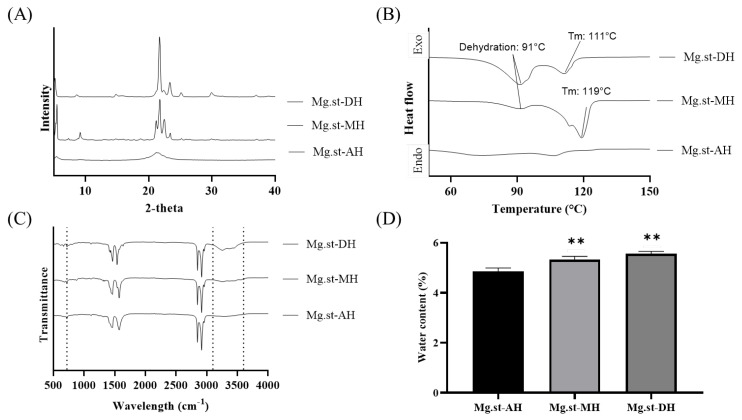
Physicochemical properties of different Mg.st hydrate forms: (**A**) XRD patterns, (**B**) DSC thermograms, (**C**) FT-IR spectrums, and (**D**) water content using Karl Fischer. ** < 0.005 compared Mg.st-AH, one-way ANOVA.

The physicochemical characteristics of the different Mg.st hydrate forms were analyzed using X-ray diffraction (XRD), DSC, FT-IR, PSD, and Karl Fischer titration. The results are shown in Figure 1. XRD analysis revealed that Mg.st-AH exhibited a broad, halo-like peak, indicative of an amorphous structure, while Mg.st-MH and Mg.st-DH showed distinct crystalline diffraction patterns. These findings were further supported by the DSC results, which showed no distinct thermal events above 150 °C. Therefore, the DSC thermogram is presented in the 50–150 °C range. While no distinct thermal event was observed for Mg.st-AH, both Mg.st-MH and Mg.st-DH exhibited endothermic dehydration peaks around 100 °C, followed by melting transitions at approximately 120 °C and 110 °C, respectively. The more pronounced endothermic peak observed for Mg.st-MH compared to Mg.st-DH suggests that Mg.st-MH possesses greater crystalline stability, which is expected to confer improved resistance to external environmental factors, such as humidity and oxidation [22,23]. FT-IR spectra showed broad absorption bands in the 3100–3600 cm⁻^1^ region, indicating the presence of structural water in the hydrates. Additionally, spectral differences near 700 cm⁻^1^ were observed, attributed to interactions between water molecules and the carboxylate groups of Mg.st. Karl Fischer titration confirmed the residual water content, showing values of 4.85 ± 0.14, 5.32 ± 0.13%, and 5.56 ± 0.09% for Mg.st-AH, -MH, and -DH, respectively. Although the differences were statistically significant due to small variability, the actual difference in residual water content was evaluated to be less than 1%. These differences are likely attributed to the presence of water molecules in their respective crystal forms, which can retain more moisture [24]. Different hydrate forms of Mg.st exhibit distinct crystalline structures, which in turn influence their physicochemical behavior and may consequently affect the performance of DPI formulations.

The particle size distributions of the magnesium stearate hydrates prior to blending were as follows: Mg.st-AH exhibited a Dv10 of 1.88 ± 0.03 μm, Dv50 of 5.03 ± 0.06 μm, and Dv90 of 14.47 ± 0.71 μm; Mg.st-MH showed a Dv10 of 1.61 ± 0.01 μm, Dv50 of 5.55 ± 0.14 μm, and Dv90 of 24.77 ± 0.97 μm; and Mg.st-DH presented a Dv10 of 1.75 ± 0.02 μm, Dv50 of 6.58 ± 0.09 μm, and Dv90 of 30.53 ± 0.99 μm. The Dv10 and Dv50 values were comparable among the three MgSt hydrates, ensuring a consistent primary particle size range for blending. Although the Dv90 values showed some variation, these differences were attributed to mild agglomeration during measurement and were not expected to persist through the blending process.

**Figure 2:**
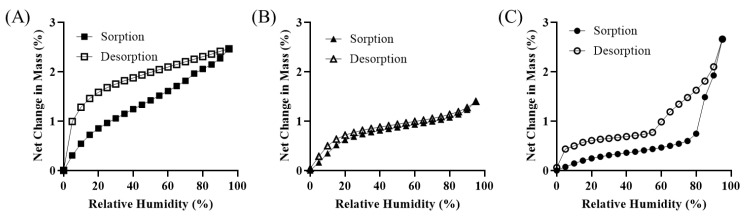
Dynamic vapor sorption: (**A**) Mg.st-AH, (**B**) Mg.st-MH, and (**C**) Mg.st-DH.

The results of the DVS analysis are shown in Figure 2. The absorption and desorption behaviors of the water molecules differed according to the hydration forms of magnesium stearate. For Mg.st-AH, a delayed desorption pattern was observed following water sorption, indicating that the absorbed water molecules were retained within the structure and released slowly. In contrast, Mg.st-MH exhibited nearly identical sorption and desorption rates, suggesting a relatively reversible interaction with moisture. Mg.st-DH showed distinct behavior: the water sorption rate increased sharply at a relative humidity above 80%, while desorption occurred rapidly down to approximately 50% RH, followed by a slower release phase. This abrupt increase in sorption at high humidity is presumed to result from a phase transition to a higher hydrate form, likely a temporary conversion to Mg St-trihydrate, leading to accelerated water uptake [25]. Among the tested forms, Mg.st-MH demonstrated the least variation under changing humidity conditions, indicating its superior stability. This suggests that monohydrate Mg.st may contribute to improved aerodynamic performance and stability in inhalation formulations.

### 3.2. Aerodynamic Performance of Arformoterol Lactose-Carrier Based Formulations Prepared with Different Mg.st Hydrate Forms

**Figure 3:**
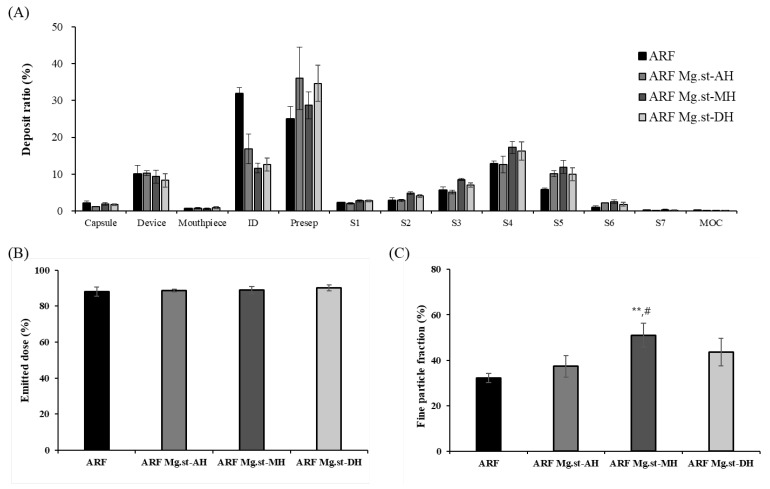
NGI results of arformoterol formulations at the initial time (n = 3): (**A**) Deposit ratio of NGI stages (**B**) Emitted dose, and (**C**) Fine particle fraction. ID, Induction port; Presep, Preseperator; S, Stage; MOC, Micro-orifice collector. ** < 0.005 compared ARF, one-way ANOVA; # < 0.05 compared ARF Mg.st-AH, one-way ANOVA.

The aerodynamic performances of the arformoterol lactose carrier-based formulations containing different Mg.st hydrate forms were evaluated using NGI, and the results are shown in Figure 3 and Table 2. Carrier-based formulations were prepared by combining arformoterol formulations with lactose carriers and Mg.st excipients, and their aerodynamic properties were analyzed. Upon comparing the ED values immediately after preparation, all formulations showed comparable values ranging from 88% to 90%, indicating minimal variation in the drug delivery output. However, the FPF differed significantly among the groups. The ARF showed an FPF of 32.16 ± 1.97%, whereas ARF Mg.st-AH, ARF Mg.st-MH, and ARF Mg.st-DH exhibited increased FPF values of 37.31 ± 4.71%, 51.02 ± 5.16%, and 43.61 ± 6.08%, respectively. Notably, the increase in FPF for the ARF Mg.st-MH was statistically significant. This enhancement is attributed to the function of Mg.st as an FCA, which reduces the adhesion between arformoterol and lactose and passivates high-energy active sites, thereby improving drug dispersion and deposition in the deep lung. Among the tested magnesium stearate forms, Mg.st-MH exhibited the highest degree of particulate interaction with the lactose surface during blending, leading to the most pronounced increase in the FPF. In contrast, the ARF Mg.st-AH and ARF Mg.st-DH formulations appeared less effective in reducing interparticle interactions compared to ARF Mg.st-MH, which may explain the higher drug concentrations observed at the induction port (ID) and pre-separator (Presp) stages. Despite showing a lower MMAD value for ARF Mg.st-AH (2.05 ± 0.01) and a simi-lar MMAD for ARF Mg.st-DH (2.24 ± 0.09) compared to ARF Mg.st-MH (2.22 ± 0.09), the observed differences in FPF may be attributed to these differences in particle interaction behavior. This enhanced interaction may have facilitated the greater detachment of arformoterol from the carrier particles during aerosolization. In tablet formulation studies, Mg.st-MH was reported to reduce tablet hardness more significantly than Mg.st-DH by interfering with the interparticulate bonding of excipients. This effect is presumed to be associated with changes in the particle size of the magnesium stearate during the blending process [18,26]. Under accelerated storage conditions (40 °C/75% RH), the FPF values of the ARF carrier-based formulations after 1 week were ARF (37.97 ± 7.15%), ARF Mg.st-AH (21.10 ± 1.78%), ARF Mg.st-MH (35.40 ± 2.09%), and ARF Mg.st-DH (30.10 ± 3.01%). After 2 weeks, the FPF values decreased to 26.62 ± 7.66%, 13.75 ± 8.81%, 33.67 ± 4.84%, and 24.18 ± 2.67% for ARF, ARF Mg.st-AH, ARF Mg.st-MH, and ARF Mg.st-DH, respectively. Despite this reduction, the drug content remained stable in all formulations, retaining approximately 100% of the labeled amount with values of 97.0 ± 0.5% for ARF, 102.4 ± 10.3% for ARF Mg.st-AH, 105.0 ± 4.1% for ARF Mg.st-MH, and 102.6 ± 2.1% for ARF Mg.st-DH. Compared to ARF without Mg.st, the formulations containing Mg.st (AH, MH, and DH) showed a more pronounced decrease in FPF over time. This phenomenon was not observed in the BUD carrier-based formulations and is considered to be specific to arformoterol. Moisture exposure under accelerated conditions likely increased the cohesiveness of arformoterol, while reduced adhesion to lactose in Mg.st-containing formulations may have promoted drug aggregation. Additional studies are required to further elucidate this observation. Among the Mg.st-containing formulations, ARF Mg.st-MH exhibited the highest FPF at both 1 and 2 weeks. This trend corresponds with the earlier DVS results, which showed that Mg.st-MH had the most stable moisture sorption profile under humid conditions. Therefore, it is likely that Mg.st-MH provides a better resistance to moisture under accelerated conditions. In contrast, Mg.st-DH may have undergone transformation into a trihydrate form owing to prolonged exposure to high humidity, leading to a continuous decline in FPF [26].

### 3.3. Aerodynamic Performance of Budesonide Lactose Carrier-Based Formulations Prepared with Different Mg.st Hydrate Forms

**Figure 4:**
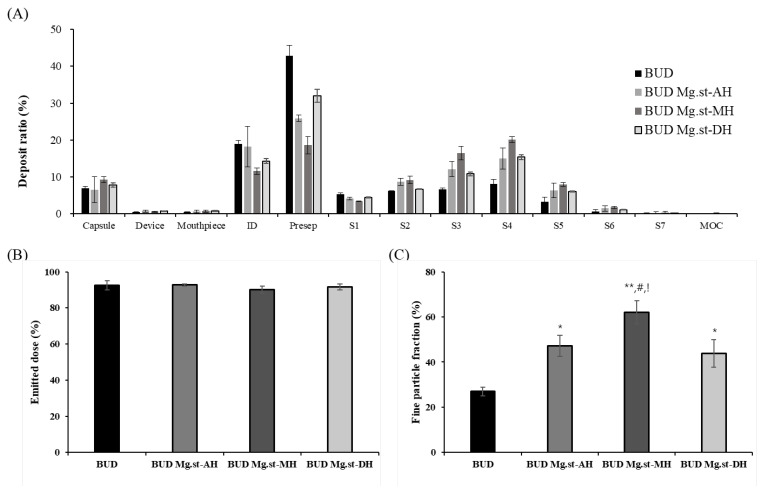
NGI results of budesonide formulations at the initial time (n = 3): (**A**) Deposit ratio of NGI stages, (**B**) Emitted dose, and (**C**) Fine particle fraction. ID, Induction port; Presep, Preseperator; S, Stage; MOC, Micro-orifice collector. * < 0.05, ** < 0.005 compared BUD, one-way ANOVA, # < 0.05, compared to BUD Mg.st-AH, one-way ANOVA, ! < 0.05, compared with BUD Mg.st-DH, one-way ANOVA.

The aerodynamic performances of budesonide lactose carrier-based formulations containing different hydration forms of Mg.st were evaluated using NGI, and the results are presented in Figure 4 and Table 3. Carrier-based formulations were prepared by blending budesonide with lactose and Mg.st excipients, and their aerodynamic properties were assessed. Similar to the arformoterol formulations, the prepared budesonide formulations initially exhibited comparable ED values across all groups. Considerable differences were observed in the FPF depending on the Mg.st types. Budesonide exhibited an FPF of 26.96 ± 3.86%, while the Mg.st-containing formulations showed significantly improved aerosolization. Budesonide Mg.st-MH exhibited the highest FPF, reaching 61.98 ± 4.09%, compared to 47.17 ± 8.11% for budesonide Mg.st-AH and 43.75 ± 1.11% for budesonide Mg.st-DH. All three Mg.st-containing groups demonstrated statistically significant improvements in FPF compared with the unmodified budesonide formulation. These enhancements are attributed to the function of Mg.st as an FCA, which modulates the interaction between budesonide and lactose carriers by reducing interfacial adhesion. Among the tested types, Mg.st-MH was particularly effective, showing the highest FPF, indicating that the monohydrate form more efficiently reduces the lactose surface energy and promotes drug detachment. This trend, consistent with the results of the arformoterol formulations, suggests that Mg.st-MH provides superior aerodynamic performance across different active pharmaceutical ingredients. Following 1- and 2-week exposure to accelerated conditions (40 °C, 75% RH), all budesonide formulations, regardless of the Mg.st type, maintained ED and FPF values close to their initial levels. Unlike arformoterol, the budesonide formulations did not exhibit a significant decline in ED or FPF after storage. This outcome appeared to be influenced by the inherent properties of each drug. Arformoterol exhibited a relatively high cohesive force, and the addition of magnesium stearate likely reduced the adhesive force, rendering it more susceptible to moisture. In contrast, budesonide, which has a stronger adhesion force, may be less sensitive to moisture because of the stable interparticulate interactions formed early in the process [21,27,28].

Among the tested forms, the incorporation of Mg.st-MH resulted in the highest inhalation efficiency of both arformoterol and budesonide. This suggests that compared to Mg.st-AH and Mg.st-DH, Mg.st-MH more effectively modulates particle interactions in lactose-based DPI formulations. Although Mg.st-MH demonstrated relatively higher accelerated stability, the overall stability appeared to be more strongly governed by the physicochemical characteristics of the drug substances themselves.

### 3.4. Raman Images of Arformoterol Formulations

**Figure 5:**
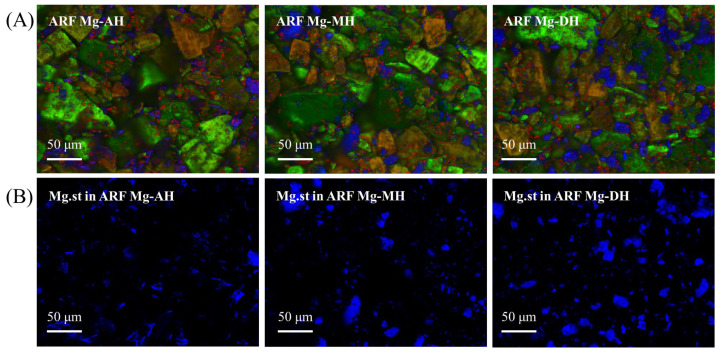
Raman images of arformoterol formulations: (**A**) Arformoterol (Red), Inhalc251 (Green), and magnesium stearate (Blue) mapped in the 2500–3000 cm⁻^1^ spectral range and (**B**) Magnesium stearate distribution in prepared arformoterol formulations.

Figure 5 presents the Raman images of the mixtures composed of arformoterol, Inhalc 251, and Mg.st hydrates. In the overlaid Raman images, arformoterol (Red), Inhalc 251 (Green), and Mg.st (Blue) were present in mixed states. Additional Raman imaging focusing solely on Mg.st was performed to evaluate the particle size distribution of the lubricant. The results of the particle size analysis of Mg.st based on Raman imaging are summarized in Table 4. Based on D_50_ values, the particle sizes of Mg.st were determined to be 7.41 ± 1.43 μm for the AH form, 6.16 ± 0.47 μm for the MH form, and 8.08 ± 1.15 μm for the DH form, respectively. Notably, Mg.st-MH exhibited the smallest particle size, with a D_50_ value more than 1 μm smaller than those of the other forms. Thus, Mg.st-MH may provide broader surface coverage within carrier-based DPI formulations of both arformoterol and budesonide, which is likely a key factor contributing to the enhanced inhalation performance observed. However, further investigation is warranted to confirm this hypothesis.

## 4. Conclusions

The use of different magnesium stearate hydrates significantly affected the aerodynamic performance of dry powder inhaler formulations. Mg.st-MH exhibited the most pronounced improvement in the FPF for both arformoterol and budesonide. This enhancement is attributed to its higher crystallinity confirmed via DSC and superior stability under high-humidity conditions, as demonstrated through DVS analysis. Furthermore, its smaller particle size and more homogeneous surface coverage, as verified via Raman imaging, likely contributed to the improved aerosolization efficiency. Notably, Mg.st-MH also maintained greater moisture stability under accelerated conditions (40 °C, 75% RH), preserving inhalation performance more effectively than the anhydrate and dihydrate forms. Thus, Mg.st-MH may be the most effective FCA for enhancing both dispersion and moisture stability in lactose-based DPI formulations. Nevertheless, further studies are warranted to validate these results under a wider range of formulation conditions.

## Figures and Tables

**Table 1:** Arformoterol and budesonide dry powder formulations with different magnesium stearate types.

Formulation	ARF	ARF Mg.st-AH	ARF Mg.st-MH	ARF Mg.st-DH	BUD	BUD Mg.st-AH	BUD Mg.st-MH	BUD Mg.st-DH
ARF	5	5	5	5	-	-	-	-
BUD	-	-	-	-	5	5	5	5
Inhalac 251	95	90	90	90	95	90	90	90
Mg.st-AH	-	5	-	-	-	5	-	-
Mg.st-MH	-	-	5	-	-	-	5	-
Mg.st-DH	-	-	-	5	-	-	-	5

**Table 2:** Emitted dose and fine particle fraction of arformoterol formulations after 0, 1-, and 2-week exposure to accelerated conditions (40 °C, 75% RH) (n = 3).

Formulation		ARF	ARF Mg.st-AH	ARF Mg.st-MH	ARF Mg.st-DH
ED (%)	Initial	87.92 ± 2.58	88.65 ± 0.71	88.86 ± 1.87	90.07 ± 1.65
	1 week	83.67 ± 3.88	95.10 ± 1.63 **	93.38 ± 1.46 **	92.70 ± 0.67 **
	2 weeks	90.17 ± 0.70	95.85 ± 2.19 **	91.82 ± 0.97 ^#^	91.57 ± 0.64 ^#^
FPF (%)	Initial	32.16 ± 1.97	37.31 ± 4.71	51.02± 5.16 **^,#^	43.61 ± 6.08
	1 week	37.97 ± 7.15	21.10 ± 1.78 **	35.40±2.09 ^#^	30.10 ± 3.01
	2 weeks	26.62 ± 7.66	13.75 ± 8.81	33.67±4.84 ^#^	24.18 ± 2.67
MMAD (μm)	Initial	2.36 ± 0.14	2.05 ± 0.01 *	2.22 ± 0.09	2.24 ± 0.09
	1 week	2.34 ± 0.04	2.36 ± 0.01	2.17± 0.09 *^,##^	2.13 ± 0.03 **^,##^
	2 weeks	2.46 ± 0.15	2.33 ± 0.06	2.23 ± 0.07	2.17 ± 0.04 *
GSD	Initial	1.65 ± 0.05	1.78± 0.09	1.80 ± 0.06	1.70 ± 0.10
	1 week	1.82 ± 0.06	1.94 ± 0.09	1.85 ± 0.07	1.87± 0.01
	2 weeks	1.77 ± 0.06	1.98± 0.11	1.84 ± 0.03	1.87 ± 0.05

* < 0.05, ** < 0.005 compared ARF, one-way ANOVA; # < 0.05, ## < 0.005 compared ARF Mg.st-AH, one-way ANOVA

**Table 3:** Emitted dose and fine particle fraction of budesonide formulations after 0, 1-, and 2-week exposure to accelerated conditions (40 °C, 75% RH) (n = 3).

Formulation		BUD	BUD Mg.st-AH	BUD Mg.st-MH	BUD Mg.st-DH
ED (%)	Initial	92.60 ± 0.49	92.72 ± 3.75	90.19 ± 0.85	91.50 ± 0.57
	1 week	93.99 ± 1.20	90.99 ± 0.53	85.51 ± 2.48 ***^,##,!!^	91.07 ± 0.14
	2 weeks	94.08±0.96	91.87 ± 1.44	90.62 ± 0.61 *^,!^	94.03 ± 0.91
FPF (%)	Initial	26.96 ± 3.86	47.17 ± 8.11 *	61.98 ± 4.09 **^,#,!^	43.75 ± 1.11 *
	1 week	23.68 ± 1.20	40.62 ± 7.82 **	60.63 ± 1.34 ***^,##,!^	47.79 ± 1.65 ***
	2 weeks	23.47 ± 3.74	47.29 ± 0.73 ***	62.27 ± 6.21 ***,^##,!!^	42.46 ± 2.75 **
MMAD (μm)	Initial	3.58 ± 0.35	2.94 ± 0.19	2.78 ± 0.05 **	2.79 ± 0.02 **
	1 week	3.61 ± 0.09	2.66 ± 0.03 ***	2.70 ± 0.005 ***	2.83 ± 0.09 ***
	2 weeks	3.77 ± 0.19	2.76 ± 0.03 ***	2.80 ± 0.10 ***	2.95 ± 0.02 ***
GSD	Initial	2.17 ± 0.13	1.92 ± 0.06 **	1.80 ± 0.07 **	1.80 ± 0.04 **
	1 week	2.14 ± 0.05	1.82 ± 0.04 **	1.78 ± 0.10 ***	1.82 ± 0.05 **
	2 weeks	2.07 ± 0.12	1.78± 0.07 **	1.71 ± 0.01 ***	1.84 ± 0.02 *

* < 0.05, ** < 0.005, *** < 0.0005 compared BUD, one-way ANOVA; # < 0.05, ## < 0.005, compared to BUD Mg.st-AH, one-way ANOVA; ! < 0.05, !! < 0.005, compared with BUD Mg.st-DH, one-way ANOVA

**Table 4:** Particle size distribution of Mg.st from Raman images (n = 3).

Formulation	ARF Mg.st-AH	ARF Mg.st-MH	ARF Mg.st-DH
D_10_ (μm)	1.58 ± 0.24	1.58 ± 0.24	1.59 ± 0.24
D_50_ (μm)	7.41 ± 1.43	6.16 ± 0.47	8.08 ± 1.15
D_90_ (μm)	20.16 ± 1.39	20.73 ± 3.39	34.39 ± 10.01
Span	2.58 ± 0.48	2.94 ± 0.51	4.02 ± 1.02

## Data Availability

The data presented in this study are available upon request from the corresponding author.

## Abbreviations

The following abbreviations are used in this manuscript:

ARF	Arformoterol
BUD	Budesonide
Mg.st	Magnesium stearate
Mg.st-AH	Magnesium stearate anhydrate
Mg.st-MH	Magnesium stearate monohydrate
Mg.st-DH	Magnesium stearate dihydrate		
DPI	Dry powder inhalation
PXRD	Powder X-ray diffraction
DSC	Differential scanning calorimetry
FT-IR	Fourier-transform infrared spectroscopy
DVA	Dynamic vapor absorption
RH	Relative humidity
FCAs	Force control agents
NGI	Next generation impactor
ID	Induction port
MOC	Micro-orifice collector
ED	Emitted dose
FPF	Fine particle fraction
